# Where Will the Next Generation of Stroke Treatments Come From?

**DOI:** 10.1371/journal.pmed.1000224

**Published:** 2010-03-02

**Authors:** D. W. Howells, G. A. Donnan

**Affiliations:** National Stroke Research Institute, Florey Neuroscience Institutes, University of Melbourne, Austin Health, Victoria, Australia

## Abstract

David Howells and G. A. Donnan discuss the next generation of stroke treatments and say that novel therapeutic targets may emerge from the stimulation of neuroplasticity and unraveling the genetic code of stroke heterogeneity.

## The Extent of the Problem

Stroke, about 80% of which is ischaemic caused by occlusion of an intracerebral artery and 20% caused by intracerebral bleeding, is the second most common cause of death and disability globally. WHO statistics indicate that stroke and other cerebrovascular diseases kill approximately 5.7 million people each year. In the United States alone it is estimated that the 780,000 symptomatic strokes detected each year may be accompanied by a further 11 million asymptomatic strokes [1]. The need to reduce this burden by better use of existing therapies and identification of new ones is pressing.

### Stroke Mechanisms and Pathophysiology: Heterogeneity Is the Key

A unique feature of stroke that creates opportunities for new therapies is the heterogeneity of its mechanisms. These range from large artery to artery embolism, cardiac embolism to *in situ* small vessel disease and even arterial dissection. Intracerebral haemorrhage may be caused by hypertensive small vessel disease, amyloid angiopathy, or rupture of saccular aneurysms. Risk factors such as atrial fibrillation, hypertension, smoking, diabetes, and disordered lipid metabolism contribute to underlying atherosclerosis or embolus formation [2]. The sequence of events, termed the ischaemic cascade, that follows an ischaemic stroke has also been established [3]. Here, neurons exposed to extreme reductions in blood flow (the “ischaemic core”) lose their membrane potential, undergo irreversible structural damage, and die. In surrounding regions (the “ischaemic penumbra”) the reduction in blood flow is sufficient to compromise neuronal function but not immediately cause neuronal death. A balance between energy supply and consumption exits and tissue survival is determined by the depth and duration of ischaemia [3],[4]. An understanding of this process has led to the concept of reperfusion and neuroprotective therapies.

### Twenty Years of Rapid but “Inherited” Advances

Interestingly, many therapeutic advances in stroke have come from research in other disciplines. For example, blood pressure lowering agents such as the ACE inhibitors, developed originally to reduce the risk of vascular injury and myocardial infarction were found to reduce stroke incidence [5],[6]. Similarly for the statins, designed to reduce LDL-cholesterol were found to protect against stroke [7]. Thrombolysis and anti-platelet therapies developed from ischaemic heart disease management [8], and hemicraniectomy to relieve pressure in some cases of ischaemic stroke was used in head trauma [9]. Even some stroke care unit management practices have come from approaches developed in cardiology, oncology, burns, and transplant medicine [10]. We have “inherited” the majority of four categories of acute and five of secondary prevention interventions with level 1 evidence of benefit in stroke since 1978 in this way (see Table 1). As this approach has been successful in the past, abandoning it now would be unwise: we suggest a continued monitoring of other disciplines, while also pursuing novel stroke-specific research. We will address the likely wins from existing classes of intervention and then speculate from where the next therapeutic classes may emerge.

**Table 1:** Acute interventions and secondary prevention strategies of proven benefit based on level I evidence.

Category	Evidence Level	Intervention	Initial or Important Study, Year [Reference]	RRR	ARR	NNT_1_
**Acute stroke**	**Proven**	Stroke unit	Langhorne et al., 1993 [10]	6.5%	3.8%	26
		Thrombolysis (tPA)	NINDS, 1995[58]	9.8%	5.5%	18
		Aspirin	IST, 1997[59]	2.6%	1·2%	83
		Decompression surgery for IS	Vahedi et al., 2007 [9]	48.8%	23%	4^a^
	**Under evaluation**	Recombinant factor 7 for ICH	Mayer et al., 2005[60]	—	—	—
		Surgery for ICH	Mendelow et al., 2005[61]	—	—	—
		Extending time window for thrombolysis	DIAS, 2005[62]	—	—	—
		Sonothrombolysis	Alexandrov et al., 2004[63]	—	—	—
		Thrombectomy	MERCI, 2005[32]	—	—	—
		Blood pressure lowering	ENOS, 2007[64]	—	—	—
		Neuroprotection	SAINT, 2006[65]	—	—	—
**Secondary prevention**	**Proven**	Aspirin	Canadian Co-op Study Group[66]	13.0%	1.0%	100
		Aspirin plus dipyridamole	Diener, 1996[67]	15.0%	1.9%	53
		Clopidogrel	CAPRIE, 1996[68]	10.0%	1.6%	62
		Anticoagulants	EAFT, 1993[69]	66.0%	8.0%	11
		Carotid endarterectomy	NASCET, 1991[70]; ECST, 1991[71]	44.0%	3.8%	26
		Blood pressure lowering	PROGRESS, 2001[72]	28.0%	4.0%	97^b^
		Cholesterol lowering	SPARCL, 2006[73]	16.0%	2.2%	220^b^
	**Under evaluation**	Angioplasty	Yadav et al, 2004[74]	—	—	—
		Thrombin inhibitors	RELY, 2009 [14]	—	—	—

The number needed to treat (NNT_1_) to prevent one stroke patient dying or becoming dependent (acute stroke), or to prevent one fatal or non-fatal stroke (secondary prevention), per year, are given. All figures are approximate and derived from previous analyses, the Cochrane database, or individual trials if these are the only data available. Modified from Donnan et al. [2],

a NNT for survival with mRS≤3.

b Calculations based on mean follow-up of 3.9 y in PROGRESS (NNT_3.9_ = 25) and median 4.9 y in SPARCL (NNT_4.9_ = 45).

ARR, absolute risk reduction; ICH, intracerebral haemorrhage; IS, ischaemic stroke; NNT, number needed to treat; RRR, relative risk reduction.

## The Next Generation of Treatments: New Twists on Existing Therapies

### Primary and Secondary Prevention

The greatest early gains are likely to come from enhancing existing strategies. Declines in stroke incidence and mortality in developed countries are most likely due to better risk factor control [11]. However, not all of the reduction in stroke mortality may have come from better blood pressure control. Benefits may have accrued from the recently recognised anti-inflammatory effects of ACE inhibitors and statins [12], and an inflammatory genesis of atherosclerosis may create opportunities for new therapeutic targets. Better recognition of atrial fibrillation (AF), the risk factor that is often overlooked in spite of its high age-specific attributable risk [13], is necessary and may lead to new opportunities. Prevention of stroke in AF with new classes of drugs such as the thrombin inhibitors is a reality [14]. With the growing impact of metabolic syndrome [15], incretin-based therapies, which help control hyperglycaemia and hyperlipidemia [16], combined with better lifestyle management may also be useful in the future. Anti-platelet agents have been a mainstay of secondary stroke prevention (Table 1). However, a ceiling may have been reached of about 20% relative risk reduction, and further anti-platelet effects may cause unacceptable bleeding [17]. Compounds with actions “beyond the platelet” need to be identified, such as thromboxane receptor antagonists.

Can imaging and other biomarkers assist in the search for new therapies? Although currently little evidence indicates that this would be cost effective, screening to detect asymptomatic aneurysms, variations in Circle of Willis anatomy [18] and arterial collateralization [19] may ultimately prove useful. For example, patients with reduced capacity to redistribute cerebral blood flow and thus maintain perfusion above ischemic thresholds are likely to be susceptible to larger strokes. Reports that variation in expression of molecules such as thrombin activatable fibrinolysis inhibitor (TAFI) [20] and plasminogen activator inhibitor-1 (PAI-1) [21] might define risk in specific stroke subsets needs evaluation in the general population. Bioinformatics may help assess panels of protein or mRNA biomarkers to assess stroke risk. Systems controlling clotting and fibrinolysis and regulating inflammation or oxidative stress may be particularly informative.

### Acute Stroke

#### Novel approaches to thrombolysis

Recanalisation and restoration of blood flow by thrombolysis with tissue plasminogen activator (tPA) benefits only a small proportion of stroke patients [22]. The narrow 3-hour time window and the risk of bleeding limit its use [23]. The time window has now been extended to 4.5 hours, and penumbral imaging with MR or CT may extend this further [24],[25]. The development of biomarker assays for stroke duration or individual risk of bleeding may increase the proportion of eligible patients. Biomarker assays may also be used to improve the toxicity profile of thrombolytic agents and help develop ways to make thrombolytics safer, for example by using the platelet-derived growth factor receptor, alpha (PDGFR-α) antagonist imatinib to reduce tPA-induced bleeding [26]. Another possibility is TAFI, with a genotype associated with stroke risk [27], circulating activity that modifies outcome after thrombolytic therapy [28] and the capacity to make clots resistant to heparins [29]. With the potential to reduce time to artery opening by up to 90 minutes [30], TAFI inhibitors might be effective and safe profibrinolytic agents for use with existing thrombolytic therapies [31].

#### Mechanical clot removal/disruption

The use of mechanical devices to remove clots after the acute stroke event is a logical approach but it is highly labour- and capital-intensive. Early recanalisation success was demonstrated with the MERCI Retriever embolectomy device [32] and has been followed by a number of others, most recently the Penumbra device [33]. While it remains to be proved that clinical benefit accrues, based on the shift from thrombolysis to more direct catheter-based intervention in acute myocardial ischemia management, a similar pattern for acute stroke is likely. Opportunities exist to develop improved clot-retrieval devices and, more importantly, health care system changes to allow deployment effectively in a timely manner.

#### Neuroprotection

Despite disappointments in the area of neuroprotection, the rhetoric “neuroprotection is dead” seems premature. Systematic reviews and meta-analyses have revealed deficiencies in experimental designs. Failure to consider bias [34] and comorbidities common in human stroke [35] all lead to over-optimistic interpretations of preclinical animal testing. A more rational approach is needed in the sequence of animal to human studies. The heterogeneity of stroke supports the breadth of preclinical evaluation recommended by the Stroke Therapy Academic Industry Roundtable (STAIR) [36], while the fundamentals of good science demand careful bias avoidance [34]. In addition it would seem desirable to have evidence that new drugs reach their hypothesised targets and elicit at least a surrogate response once there. Hypothermia does tick all the appropriate preclinical boxes [37] and has been found to be effective in protecting against the neurological sequelae of cardiac arrest [38]; a large-scale Phase III trial in stroke is needed in spite of logistical difficulties. Also, compounds that directly depress body temperature should perhaps be considered. For example, improgan, a member of a new class of nonopioid analgesics, can reduce core temperature in rodents by 1°C within 10 minutes [39], and hydrogen sulphide can rapidly induce a suspended animation-like state [40]. Drugs that alter the thermoregulatory set-point and make hypothermia more tolerable by reducing shivering are already being considered [41]. We should also entertain alternative mechanisms of action of hypothermia such as control of oedema and local compression.

Timing of treatment following stroke is critical. It may be that neuroprotection is of value only if reperfusion ultimately occurs. In other disciplines, graft ischaemia times for renal, cardiac, and lung transplantation due to developments in effective cold storage and preservative fluids are impressive. Hence “freezing” the penumbra with neuroprotectants may be a realistic goal while waiting for reperfusion. An example is with normobaric oxygen, which increases penumbral oxygen partial pressure and reduces infarct volume in animals [42]. Prolongation of penumbral survival has been inferred in Phase II MR-based studies and pilots of therapy performed in humans [43].

## Where May the Next New Class of Therapies Come From?

Although a number of avenues of research may bring rewards in terms of completely new classes of intervention for the prevention and treatment of stroke, we believe two areas are most likely to generate completely new classes of therapeutic targets.

### Stimulation of Plasticity

One of the most important advances in neuroscience has been the recognition of activity-dependent plasticity. Synapses, the base units of connectivity, form and disappear depending upon activity and experience, and axons and dendrites can reach out to and withdraw from new targets [44]. Although it had become established that astrocytes and microglia respond rapidly to injury, the realisation that new neurons and supporting oligodendroglia could be generated from pools of neural stem cells and progenitors was a paradigm shift in neurology [45],[46]. These processes offer new and exciting therapeutic opportunities. At the simplest level, we can use observations of benefit after enriching the environment or increasing motor activity to improve traditional rehabilitation strategies [47]. Effective delivery of growth-promoting factors, including the nerve growth factor and glial cell line-derived neurotrophic factor families of proteins, to enhance plasticity and regeneration may also prove effective. Alternatively, mobilization and activation of endogenous neurogenesis/plasticity with drugs such as granulocyte colony-stimulating factor (G-CSF) may be attractive [48]. Although stem cell implants into the brain may currently deliver only a supportive or plastic environment that aids recovery, these cells can mature and integrate into the host neural circuitry [49]. Given that repopulation of connective tissue scaffolds by stem cells can reconstitute a beating animal heart [50], the same may ultimately be possible for regions of damaged brain.

Importantly, evidence is emerging that neural recovery and immune function are intimately linked [51]. Neural outputs seem to regulate bone marrow and spleen activity, while cytokines and related molecules act both locally and systemically to facilitate neuroimmune communication. While this interaction provides considerable scope for clinical intervention, for example by using G-CSF to mobilise neural stem cells [48] or manipulating microglial or macrophage mediated axonal plasticity [52], it is a double-edged sword. Many of the candidate molecular targets directly influence both acute injury development and later neurovascular remodelling [53]. Our approaches need to be sophisticated enough to deal with these critical temporal issues.

### Unravelling the Genetic Code to the Heterogeneity of Stroke

There is about a 40% gap in the population attributable risk for stroke when all known risk factors are considered [54]. Much of the gap may consist of genetic contributions to risk in some form. Fortunately, there have been enormous advances in the genetics of stroke. Initial linkage analysis studies identified, rare conditions such as CADASIL (cerebral autosomal dominant arteriopathy with subcortical infarcts and leucoencephalopathy) due to mutations in the *NOTCH3* gene [55], which is involved in cell signalling and fate during embryonic development. Subsequently, a candidate gene approach using case-control designs produced a large number of potential gene polymorphisms, many of which could not be replicated and were probably the product of underpowered studies. The emergence of the concept of polygenic contributions to the stroke syndrome, gene chip technology and genome-wide association studies (GWASs) has revolutionized the area. Large international cooperative studies with sample sizes in the thousands have enabled investigators to produce reliable data. For example, by genotyping more than 310,000 single-nucleotide polymorphisms (SNPs) in more than 1,700 intracranial aneurysms and 7,400 controls, SNPs on Chromosomes 2q, 8q, and 9p were associated with aneurysmal presence. The biological implications come from an understanding of the function of these genes as our research effort explores their biology. Chromosomes 8q and 9p both have genes that are associated with progenitor cells and expressed in blood vessels. The main candidate gene on 8q is *SOX17*, which is required for endothelial formation and maintenance [56]. The implications for the development of gene-based or other therapies are obvious. Similarly, investigators of the International Stroke Genetics Consortium found an association between SNPs in the Chromosome 9p21.3 region and large-artery stroke [57]. GWASs are still in their infancy and are dependent on careful phenotyping and large sample sizes. However, the likelihood that completely novel therapeutic classes emerge from these studies is extremely high.

## Summary

Remarkable progress has occurred over the last two decades in stroke interventions. Many have been developed on the basis of their efficacy in other disorders. This “inheritance” approach should continue, but two areas where completely novel therapeutic targets might emerge are the stimulation of neuroplasticity and unraveling the genetic code of stroke heterogeneity (Table 2). For the former, the next steps are to identify small-molecule, nontoxic compounds that most effectively enhance plasticity in animal models, and then subject them to clinical trial in humans. For the latter, more and larger-scale cooperative GWASs in carefully phenotyped stroke populations are required to better understand the polygenic nature of cerebrovascular disease. Then, the physiological relevance of genetic abnormalities can be determined in in vitro and in vivo systems before candidate compounds are developed.

**Table 2:** Five key papers in the field of stroke.

Advance	Paper and Title	Importance	Ref.
**Understanding risk factors leads to drug development.**	**Connolly et al., 2009:** Dabigatran versus warfarin in patients with atrial fibrillation.	Prevention of stroke in AF with new classes of drugs such as the thrombin inhibitors is important because of the high age-specific attributable risk of AF.	[14] H
	**Brouns et al., 2009:** Carboxypeptidase U (TAFIa) decreases the efficacy of thrombolytic therapy in ischemic stroke patients.	Evidence is emerging that genotype and activity of TAFI both is associated with stroke risk and interferes with the efficacy of tPA. TAFI inhibitors have the potential to significantly reduce clot lysis time.	[28] H
**Robust neuroprotection is possible and needs to be trialled.**	**van der Worp et al., 2007:** Hypothermia in animal models of acute ischaemic stroke: a systematic review and meta-analysis.	The data supporting the use of hypothermia for neuroprotection in animal models of stroke are robust; hypothermia is now routinely used to prevent the neurological consequences of cardiac surgery. With the possibility of effective pharmacological control of shivering, a large-scale trial of hypothermia in stroke is overdue.	[37] A
**Damage and repair go hand in hand.**	**Lo, 2008:** A new penumbra: transitioning from injury into repair after stroke.	This seminal paper takes a holistic view of stroke pathophysiology and argues that many of the highly conserved “deleterious” mediators of acute injury are also essential for later plasticity and recovery. Our therapeutic approach to preserving the ischemic penumbra needs to take this complexity into account.	[53] ^A+H^
**Population genetics will identify new risk factors and therapeutic targets.**	**Gschwendtner et al., 2009:** Sequence variants on Chromosome 9p21.3 confer risk for atherosclerotic stroke.	This large multinational GWAS revealed that Chromosome 9p21.3 represents a major risk locus for atherosclerotic stroke. This region contains a number of candidate genes.	[57] H

A animal study.

H human study.

## Abbreviations

AF: atrial fibrillation
CADASIL: cerebral autosomal dominant arteriopathy with subcortical infarcts and leucoencephalopathy
G-CSF: granulocyte colony-stimulating factor
GWAS: genome-wide association study
PAI-1: plasminogen activator inhibitor-1
PDGFR-α: platelet-derived growth factor receptor alpha
SNP: single nucleotide polymorphism
TAFI: thrombin activatable fibrinolysis inhibitor
tPA: tissue plasminogen activator
